# Experimental Behavior of Cracked Reinforced Concrete Columns Strengthened with Reinforced Concrete Jacketing

**DOI:** 10.3390/ma13122832

**Published:** 2020-06-24

**Authors:** Ahmed Mohamed Sayed, Mohamed Mohamed Rashwan, Mohamed Emad Helmy

**Affiliations:** 1 Department of Civil Engineering, College of Engineering, Assiut University, Assiut 71511, Egypt; mohamed.rashwan@aun.edu.eg (M.M.R.); memad2661@gmail.com (M.E.H.); 2 Department of Civil and Environmental Engineering, College of Engineering, Majmaah University, Al-Majmaah 11952, Saudi Arabia

**Keywords:** RC column, RC jacketing, strengthening, column cracking, ACI-318 model

## Abstract

Reinforced concrete (RC) columns often need to be strengthened or rehabilitated to allow them to carry the loads applied to them. In previous studies, RC columns have been strengthened by jacketing, without considering the occurrence of cracking. In this study, the behavior of RC columns strengthened externally by jacketing after cracking is analyzed. The accuracy of the existing models was verified by analyzing the performance of fifteen RC columns with different cross-sections to determine the effect of new variables, such as the column size, amount of steel reinforcement, and whether the column was cracked or not, on the effectiveness of strengthening. The analysis demonstrated that this strengthening technique could effectively improve both the ductility and strength of RC column cross-sections. The results indicate that the model suggested by the ACI-318 code can predict the ultimate load capacity of RC columns without strengthening, or strengthened by RC jacketing before or after cracking, with higher accuracy and material efficiency. The RC columns without strengthening met the safety limit of the ACI-318 model. However, for strengthened columns, a reduction coefficient must be used to enable the columns to meet the safety limit, with values of 94% and 76% for columns strengthened before and after cracking, respectively. Furthermore, strengthening after cracking affects the ultimate load capacity of the column, with 15.7%, 14.1%, and 13.5% lower loads for square, rectangular, and circular columns than those strengthened before cracking, respectively.

## 1. Introduction

Reinforced concrete (RC) columns must always be strengthened or rehabilitated to improve their efficiency in carrying the loads affecting them. The efficiency of columns in bearing loads can be affected by increases in the external loads, the generation of additional loads, such as seismic loads, or defects in the column due to mistakes in the design phase, the use of insufficient materials in the concrete mixing, or corrosion in the steel reinforcements of RC columns.

Many methods of strengthening RC columns have been developed. Older methods include the use of RC or steel angle jacketing, and modern methods include the use of different types of fiber-reinforced polymer jacketing. These methods can all increase the efficiency of RC columns by increasing their ultimate load capacity. Many previous studies have focused on strengthening RC columns under the influence of seismic loads by different methods [1,2,3,4,5,6,7,8,9]. Many studies have also studied the strengthening of RC columns under the influence of axial [10,11,12,13,14,15,16,17,18,19,20,21,22,23,24,25,26] or flexural [27,28,29,30,31,32] loads, or both [33,34,35,36,37,38]. Recently, a review was conducted that found that approximately 99 studies have been conducted on the strengthening of RC columns in the last twenty years [39]. The review was structured according to the method of strengthening, and indicated that none of these studies considered the effect of the presence of a preload or pre-cracks in the RC columns on the strengthening process. Some numerical studies considered the effect of preloading on strengthening with steel angle jacketing [40,41,42], and demonstrated that preloading greatly impacts the efficiency of the strengthening of RC columns according to the preload on the column. Therefore, in this study, RC columns were strengthened with RC jacketing after cracks formed on the sides of the columns, to determine their effect on the efficiency of strengthening.

Many of the current design models proposed by existing design codes [43,44] or previous research [23,45] can predict the ultimate load capacity with high accuracy, if there are no cracks or preloads on the RC columns. However, when cracks and preloads are present, these models require reduction factors to enable the design to fall within the safety limits [40].

The presence of preloads on the RC columns can cause cracking. Therefore, the strengthening process must at least consider the presence of loads that cannot be removed from the columns during the strengthening process. Consequently, in this study, an experimental test was conducted to assess the strengthening of columns by RC jacketing with different proportions of longitudinal steel reinforcements, to determine the effects of the presence of cracks in RC columns with different cross-sections on the efficiency of the strengthening process. The experimental results were compared to the existing design codes in order to determine their accuracy in such cases, as well as the values of the reduction factors required to allow the design to meet the safety limits.

The strengthening of RC columns, regardless of whether cracks have formed or not, aims to improve the strength, efficiency, and stiffness of RC columns, thereby maximizing their load capacity. Therefore, the purpose of this study—after reviewing previous research—is to address the shortcomings in the process of estimating the maximum axial load capacity of RC columns using RC jacketing after the appearance of cracks resulting from preloading. This study focuses on the influence of several variables that may have an impact on the efficiency of the strengthening process, such as the column size, amount of steel reinforcement, and whether the column is cracked or not. All of these variables aim to observe the extent to which these cracks affect the efficiency of the strengthening process. If there is a difference between the strengthening process of whether cracks have formed or not, the necessary reduction coefficient should be determined, which can be applied when using the current design codes.

## 2. Experimental Tests

### 2.1. Specimen Details

**Figure 1:**
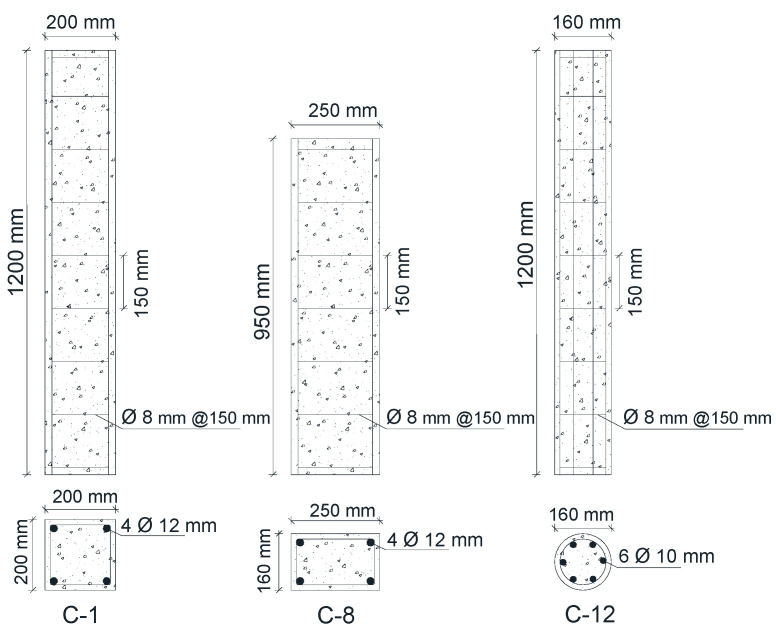
Geometric details of the RC columns.

Fifteen RC columns with different cross-section shapes, divided into three groups, were tested under static loads. The first group consisted of five RC columns with a 200 mm × 200 mm square cross-section and a total height of 1200 mm. The second group consisted of five RC columns with a rectangular cross-section of 160 mm × 250 mm and a total height of 950 mm, and the third group consisted of five RC columns with a circular cross-section of 160 mm in diameter and a total height of 1200 mm, as shown in Figure 1. A column from each group was tested until the point of failure and used as a control specimen. A load was then applied to the remaining columns of the group until the first crack appeared, following the approach described in Section 2.2, and the load application then ceased. The concrete compressive strength *f*_c_′ for each column is shown in Table 1. Two types of steel bars were used to reinforce the RC columns, including deformed steel bars with diameters of 10 mm and 12 mm, which were used for vertical reinforcement, and plain steel bars with a diameter of 8 mm, which were used for stirrup reinforcement. All RC column stirrups had a diameter of 8 mm and spacing of 150 mm, and had a yield and ultimate strengths of 327 and 457 MPa, respectively, as shown in Figure 1. The yield strengths of the bars with diameters of 10 mm and 12 mm were 411 MPa and 441 MPa, and the ultimate strengths were 580 MPa and 603 MPa, respectively.

**Figure 2:**
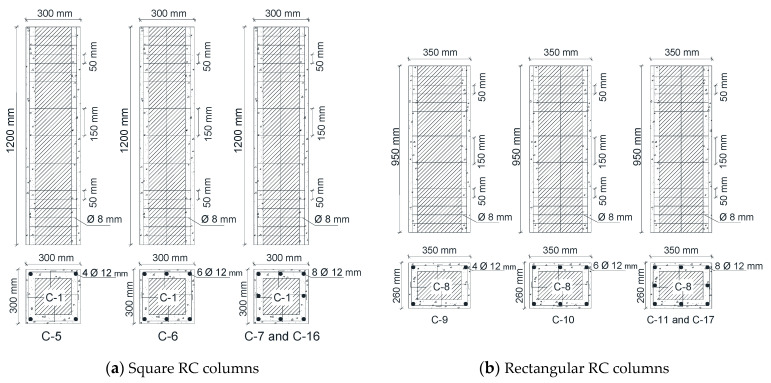
Details of the RC columns strengthened by complete RC jacketing.

**Figure 3:**
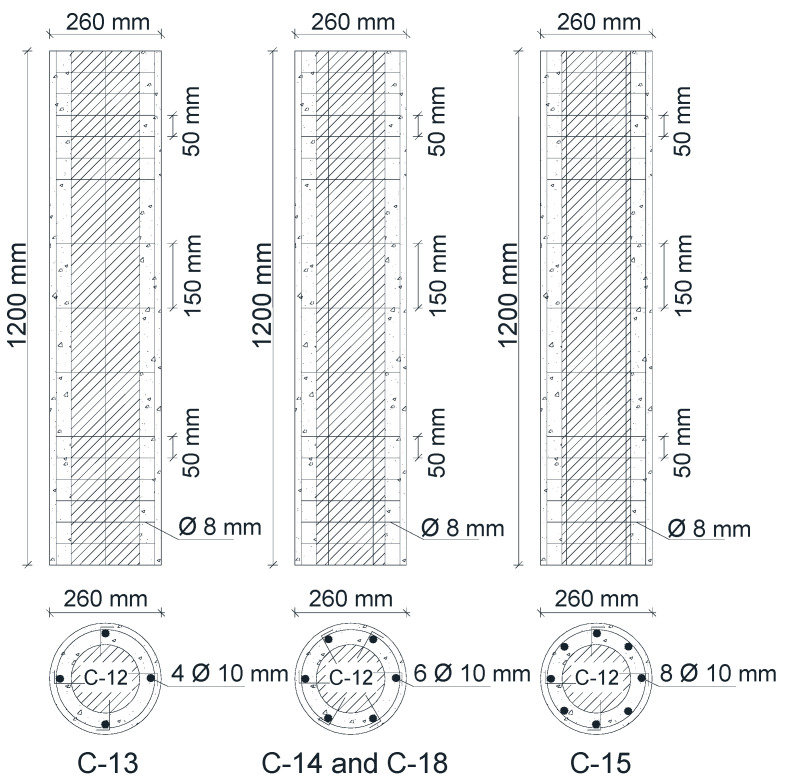
Details of the circular RC columns strengthened by complete RC jacketing.

The three specimens that were tested until the point of the first crack were strengthened by complete reinforced concrete jacketing. This involved increasing the concrete cross-section dimensions for each side of the square and rectangular columns by 100 mm, as shown in Figure 2, and increasing the diameter of the circular column by 100 mm, as shown in Figure 3. The compressive strengths *f*_c_′ of the concrete jacketing and steel reinforcements used to strengthen each column are listed in Table 2.

The strengthening process is carried out in several steps: First, the external surfaces of all the columns that are to be strengthened are cleaned and have their surfaces roughened by removing the outer concrete cover and any unstable concrete parts that have resulted from cracks. Second, the holes that are made in the sides of the column’s perimeter with a depth of 50 mm and a diameter of 8 mm are distributed on the sides of the column’s cross-section, as shown in Figure 2 and Figure 3. Then, the anchorage bolts are placed in the form of an L-shape with a total length of 90 mm and a diameter of 5 mm, and are fixed inside these holes using Kemapoxy adhesive material 165. Third, new stirrups are added with a diameter of 8 mm with the distribution, shown in Figure 2 and Figure 3, and they are connected to the pre-fixed anchorage bolts, then added to the new vertical steel reinforcement inside these stirrups. Finally, new concrete is cast to be the external strengthening of the columns.

### 2.2. Instrumentation and Test Setup

All of the RC columns were tested using a loading machine (Civil Engineering Department Lab, Assiut University, Assiut, Egypt) with a capacity of 5000 kN. The testing machine had upper and lower heads. The upper head could be fixed at any height according to the column length, while the lower head only moves upward when loading. A computer-aided data acquisition system was used to monitor the load, displacements, and strains throughout the loading tests at selected time intervals. The vertical displacement was measured to monitor the axial shortening of each column and the horizontal displacement of the column at mid-height; the strain on the vertical steel reinforcement, and the horizontal strain on the concrete surface at the mid column height were also measured. Electrical resistance strain gauges were attached to the steel reinforcements and concrete surfaces to measure the strain of the steel reinforcements and concrete. The displacement was measured using linear variable displacement transducers (LVDTs).

## 3. Results and Discussion

### 3.1. Crack Patterns and the Axial Load Capacity of Control Columns

**Figure 4:**
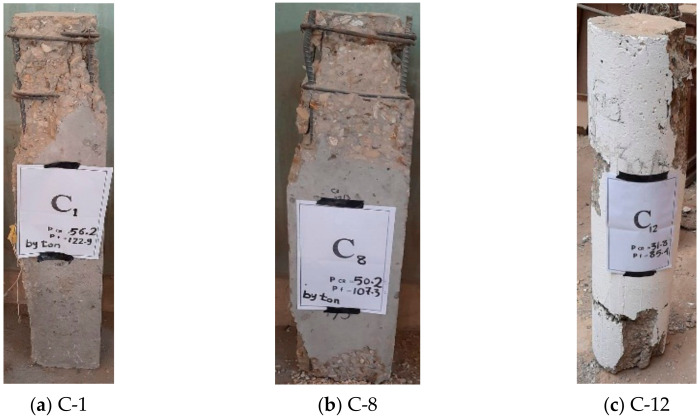
Crack patterns of the control RC columns without strengthening.

The first cracks in the control columns (C-1, C-8, and C-12) were observed at the bottoms and tops of the columns at loads of 562 kN, 502 kN, and 318 kN, respectively. By increasing the applied load, the cracking increased and the final failure location was observed at the top of the column height, as shown in Figure 4. The control RC column test specimens failed at applied loads of 1229 kN, 1073 kN, and 635 kN, respectively.

**Figure 5:**
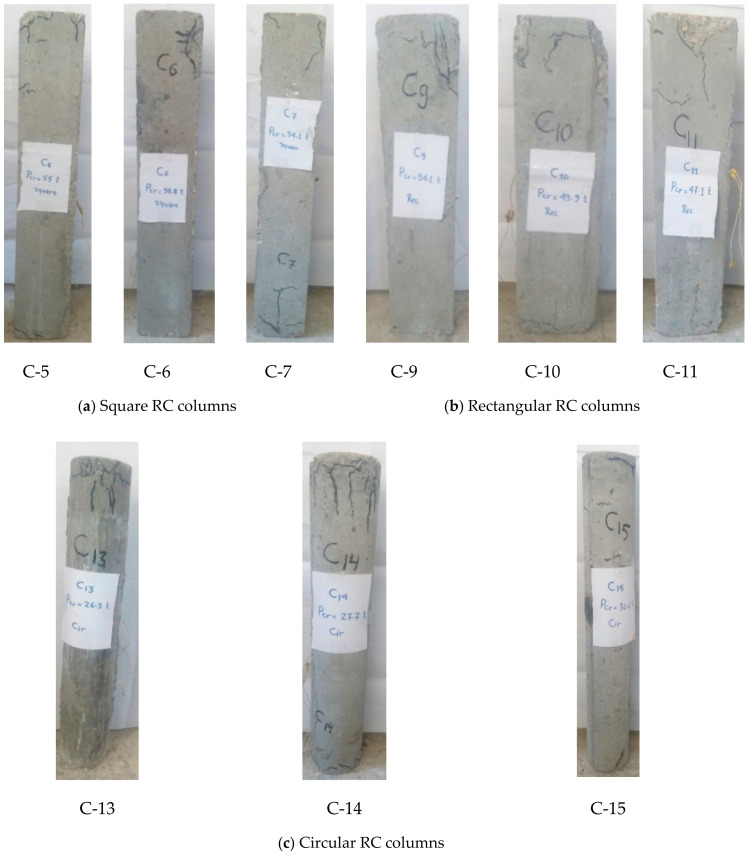
Crack patterns of the RC columns without strengthening.

For the RC columns strengthened by complete RC jacketing, the first cracks were observed at the bottoms and tops of the columns, as shown in Figure 5. After reaching the first crack load, the load was stopped until the column was set for strengthening. Table 3 shows the cracking and ultimate load capacities of the control RC columns without strengthening, the RC columns strengthened without pre-cracking, and the RC columns that will be strengthened by complete RC jacketing after cracking.

### 3.2. Crack Patterns and the Failure Loads of the Strengthened Columns

**Figure 6:**
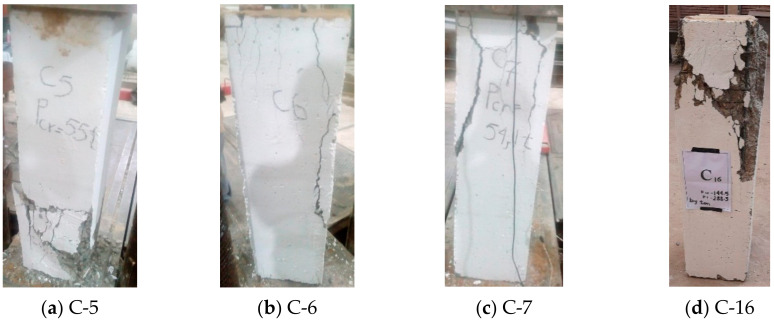
Crack patterns of the strengthened square RC columns.

Two types of strengthening were tested, i.e., strengthening after cracking (C-5, C-6, and C-7) and strengthening before cracking (C-16). The shapes of the cracks and the failure in almost all columns were similar. A major vertical crack parallel to the axis of the column was observed and identified as the reason for the failure of the square RC columns, as shown in Figure 6.

**Figure 7:**
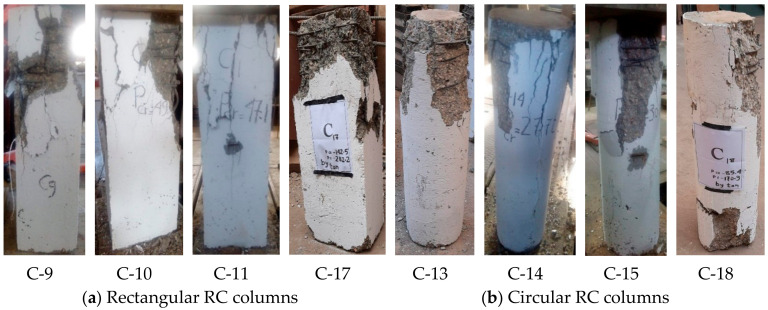
Crack patterns of the strengthened RC columns.

Furthermore, for the rectangular and circular columns that were strengthened before and after cracking, the shape of the failure was identical to that of the square columns, as shown in Figure 7.

The ultimate load of all of the columns was determined from the measured variables, as shown in Table 4. The table indicates that the columns without strengthening exhibited the lowest ultimate load values, and the columns strengthened without cracks exhibited the highest load values. The load values of the columns strengthened after cracking were between these. Furthermore, the efficiency of the strengthening process for the rectangular columns was better than that for the square columns, as the percentage increase in the failure load of the rectangular columns after strengthening was greater than that in the square columns with the same cross-sectional area and strengthening process. Furthermore, the axial load capacity of the columns that strengthened after cracking were lower than those of the columns strengthened before cracking, and these deficiencies changed with the shape of the column cross-section, with decreases of 15.7%, 14.1%, and 13.5% for the columns with square, rectangular, and circular cross-sections under the same strengthening conditions, respectively.

### 3.3. Load–Displacement Relationships

**Figure 8:**
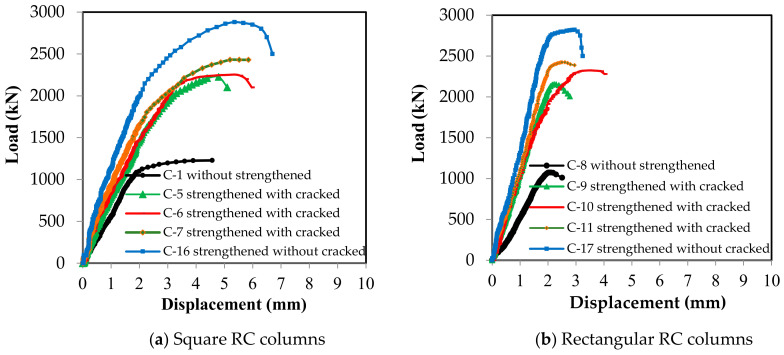

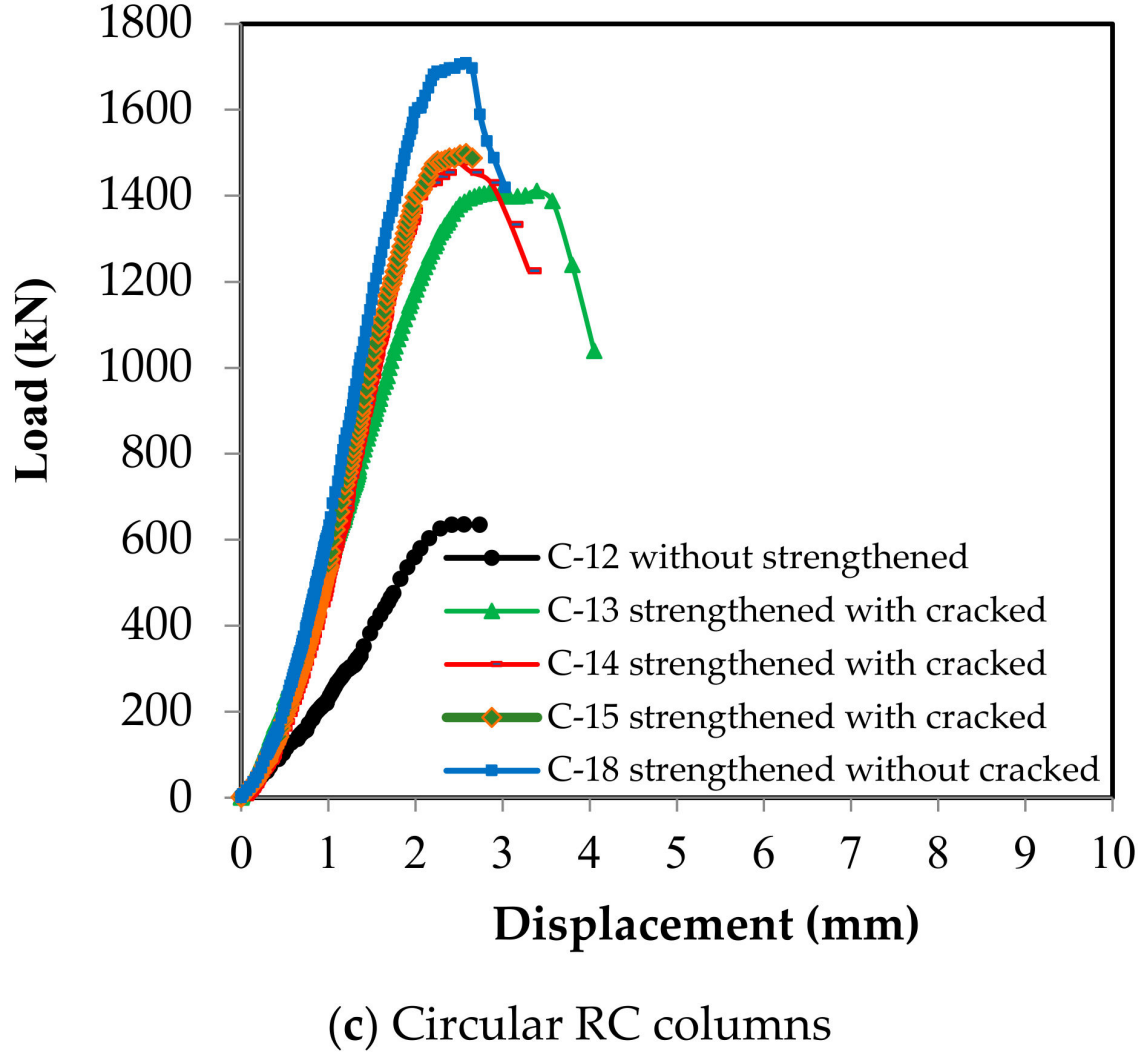
Load–displacement relationships for the RC columns.

The vertical displacement of the columns when a load was applied until the point of failure was measured, as shown in Figure 8. These Figures indicate that the strengthened columns exhibited greater displacement than the strengthened columns at the same applied load, and that the efficiency of the columns strengthened before cracking was greater than that of the columns strengthened after cracking. This is evident from the angle of inclination of the curves. The inclination of the curves also indicates the differences between the columns with different cross-sections. The vertical displacement values were close for all columns, regardless of whether they were strengthened, as these values correspond to the maximum strain of concrete and the yield strain of the main vertical steel reinforcement.

### 3.4. Effect of Longitudinal Steel Reinforcement in RC Jacketing

**Figure 9:**
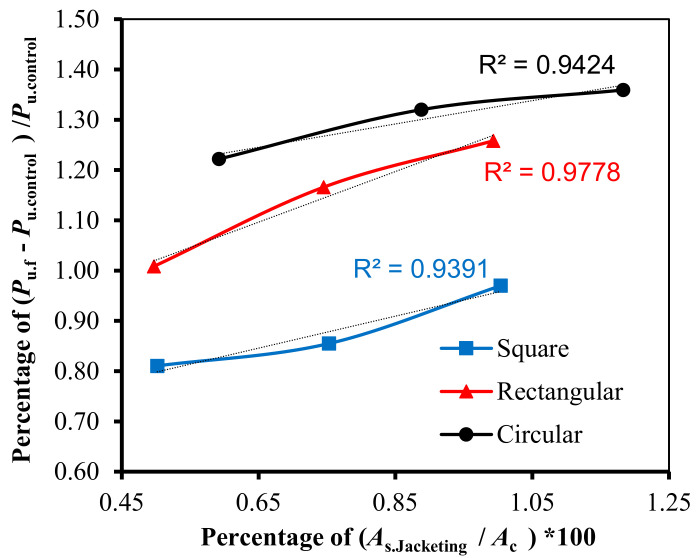
The relationship between the percentage of increase in column capacity and the percentage of longitudinal reinforcement in RC jacketing for different column cross-section shapes.

Figure 9 indicates that the effect of the longitudinal steel reinforcement in the RC jacketing is very similar among all of the RC column cross-section shapes, where the correlation coefficients are 0.97, 0.99, and 0.97 for the square, rectangular, and circular RC columns, respectively. This result corresponds to the existing models, which calculate the steel reinforcement effect without any reduction coefficients. That is, the effect of longitudinal reinforcement is linear when increasing the ultimate load capacity of the RC columns.

**Figure 10:**
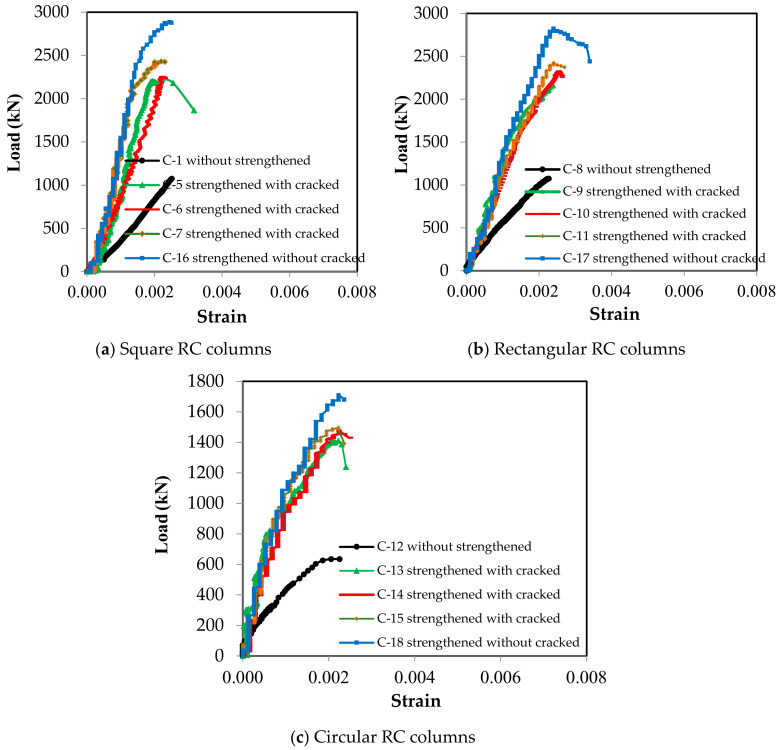
Load–longitudinal steel strain relationships for the RC columns.

Figure 10 also indicates that the relationship between the strains and loads for longitudinal steel is almost linear, and this is consistent with the result shown in Figure 9. Although the strain values decreased as the ratio of the longitudinal steel reinforcement in the RC column increased under the same load level, the maximum strain value for all of the RC columns with different ratios of longitudinal steel reinforcement reached the final yield stage of the steel material, and the failure of the RC column subsequently occurred. This is consistent with the current design models.

## 4. Comparison of the Results with the Existing Design Models

One of the most important design equations used in calculating RC column capacity is the ACI-318 code model [43], as it is the basic equation for all existing design models. The model proposed by the ACI-318 code [43] is used for the RC columns that are not strengthened or have been strengthened by RC jacketing, and gives the designed axial load capacity of the RC columns, as shown in Equation (1).

(1) $$\phi P_{n,\max}=0.80\phi \left[0.85\right.f_{c}^{\prime }(A_{g}-A_{st})+(f_{y}A_{st})]$$

Equation (1) contains more than one safety factor to reduce the load from the failure load to the design load, i.e., *ɸ*, 0.85*f′*_c_ and 0.80. If these factors are omitted, a model for calculating the ultimate axial load capacity for the RC column is obtained.

(2) $$P_{u.f}=f_{c}^{\prime }(A_{c}-A_{st})+(f_{y}A_{st})$$

**Figure 11:**
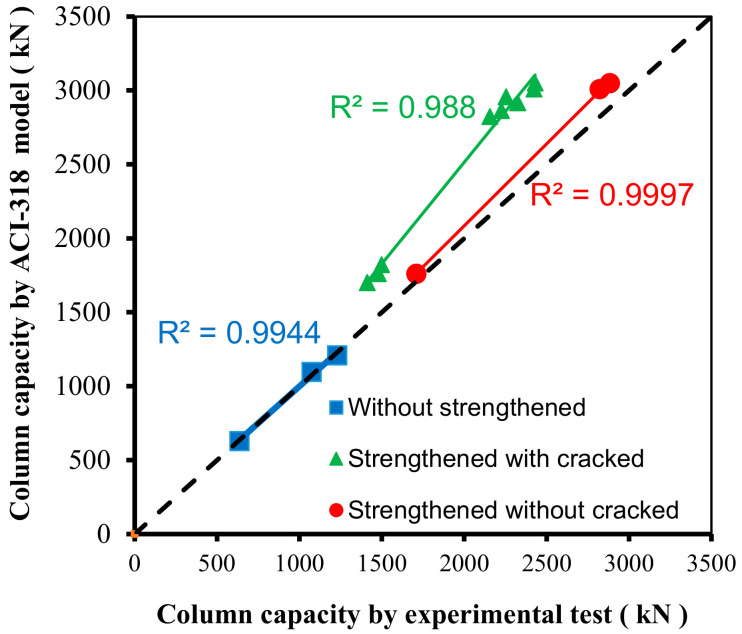
Comparison between the ACI-318 model [43] and the experimental test results.

Table 5 indicates the value expected by the ACI-318 code [43], and the ratio between this value and the experimental ultimate load values, for all columns. This is also graphically represented in Figure 11. The results indicate that the prediction of the ACI-318 code model [43] in the RC columns without strengthening was highly accurate, where the average ratio of *P_u_*_.Ex_/*P*_u.ACI_ was 1.00, with a coefficient of variation of 2.12% and a correlation coefficient of 0.997. The average ratio of *P*_u.Ex_/*P*_u.ACI_ for the load prediction for the strengthened RC columns without cracks was 0.95, the coefficient of variation was 1.82%, and the correlation coefficient was 0.998. However, the prediction values exceeded the experimental test results, and they were outside the safety limits. For the RC columns strengthened after the cracks occurred, the average ratio of *P*_u.Ex_/*P*_u.ACI_ was 0.80, the coefficient of variation was 3.51%, and the correlation coefficient was 0.994. However, all values for these columns were in the unsafe region of the predicted failure loads, as shown in Figure 11. The load predicted using the ACI-318 [43] equation should be reduced to a ratio of at least 80%, and it is preferable that the lowest ratio that was observed in the analysis process of 76% be taken to ensure that all values are within the limits of safe design.

Table 5 also shows the deficiencies between the test results and the values predicted by the ACI-318 model [43] due to the type of strengthening. Deficiencies were observed for strengthening before cracking as there were two grades of concrete in the column, as the core of the column was weak, and the RC jacketing was strong, resulting in differences in the strains of the contact surfaces between the column and reinforcement. This affected the ultimate capacity of the column, and the average ratio decreased to 5%. For strengthening after cracking, the deficiency was the result of two factors. One factor was the difference in strains between the concrete in the core of the column and the concrete used for strengthening. The second is the presence of cracks in the core of the column, which affected the efficiency of the strengthening and the ultimate capacity of the column. The average percentage of deficiency reached 20%.

## 5. Conclusions

RC columns are commonly strengthened by RC jacketing; however, all previous studies and models used to calculate the ultimate load capacity of these columns were conducted on columns that did not contain cracks before strengthening. Therefore, the main objective of this research was to determine the efficiency of the strengthening of such columns after crack formation. The results were compared to the model available in the ACI-318 code [43] in order to determine the accuracy of its use in such cases. From the analysis of the results, some conclusions were drawn, as follows:

- The presence of cracks before strengthening affects the maximum load capacity of RC columns. The capacities of square, rectangular, and circular RC columns strengthened after cracking were 15.7%, 14.1%, and 13.5% lower than those of columns strengthened before cracking, respectively.
- The ACI-318 model [43] can predict the ultimate load capacity with higher accuracy and higher material efficiency. However, it requires reduction factors of up to 0.94 and 0.76 when applied to columns strengthened without and with cracks, before all RC columns are considered safe, respectively.
- The effect of the ratio of vertical steel reinforcement in the RC jacketing on the ultimate load capacity of the RC columns is linear.
- The effect of the vertical steel reinforcement ratio in the RC jacketing on the ultimate load capacity of the RC column is linear, regardless of whether the column was strengthened before or after cracking.

Future research could focus on the study of strengthening RC columns after cracks that occur under the effect of preloading using other types of strengthening operations, such as the use of fiber-reinforced polymer (FRP) composite or the use of steel angle jacketing, which does not receive considerable attention in the literature. All of these new research findings guide the current code to complete the existing design gaps.

## Figures and Tables

**Table 1:** Properties of the reinforced concrete (RC) column specimens analyzed in this study.

Column Shape	Column Specimens	Concrete Dimension (mm)			*A*_s_	*A*_s_/*A*_c_	Concrete *f_c_*’ (MPa)
		*b*	*t*	*h*			
Square	C-1, 5, 6, 7 and 16	200	200	1200	4Ø12	1.13%	25.5
Rectangular	C-8, 9, 10, 11 and 17	160	250	950	4Ø12	1.13%	22.7
Circular	C-12, 13, 14, 15 and 18	160 Diameter		1200	6Ø10	2.34%	22.1

**Table 2:** Properties of the RC column specimens strengthened by complete RC jacketing.

Column Shape	Column Specimens	Initial Concrete Dimensions (mm)				RC Jacketing					
		*b*	*t*	*h*	*A_s_*	Concrete *f*_c_’ (MPa)	Thickness (mm)	New Dimension (mm)		*A*_sj_	*A*_s_/*A*_c_
								*b*	*t*		
Square	C-16	200	200	1200	4Ø12	29.4	50	300	300	8ø12	1.50%
	C-5									4ø12	1.00%
	C-6									6ø12	1.25%
	C-7									8ø12	1.50%
Rectangular	C-17	160	250	950	4Ø12	30.2	50	260	350	8ø12	1.50%
	C-9									4ø12	1.00%
	C-10									6ø12	1.24%
	C-11									8ø12	1.50%
Circular	C-18	160 Diameter		1020	6Ø10	28.9	50	260 Diameter		6Ø10	1.78%
	C-13									4Ø10	1.48%
	C-14									6ø10	1.78%
	C-15									8ø10	2.07%

**Table 3:** Summary of the experimental test results.

Column Shape	Column Specimen	Cracking Load (kN)	Ultimate Load (kN)
Square	C-1	562	1229
	C-16	1445	2883
	C-5	550	-
	C-6	588	-
	C-7	541	-
Rectangular	C-8	502	1073
	C-17	1425	2822
	C-9	541	-
	C-10	499	-
	C-11	471	-
Circular	C-12	318	635
	C-18	854	1709
	C-13	263	-
	C-14	277	-
	C-15	301	-

**Table 4:** Summary of RC columns strengthened by RC jacketing.

Column Shape	Column Specimen	Ultimate Load (kN)	% Increase	% Decrease
Square	C-1	1229	---	---
	C-16	2883	134.5	---
	C-5	2225	81.0	---
	C-6	2253	83.3	---
	C-7	2430	97.7	15.7
Rectangular	C-8	1073	---	---
	C-17	2822	163.0	---
	C-9	2155	100.8	---
	C-10	2324	116.6	---
	C-11	2423	125.8	14.1
Circular	C-12	635	---	---
	C-18	1709	169.1	---
	C-13	1411	122.2	---
	C-14	1479	132.9	13.5
	C-15	1498	135.9	---

% Increase = percentage of increase in the failure load from the RC column without strengthening. % Decrease = percentage of decrease in the failure load from the RC column strengthened before cracking.

**Table 5:** Summary of the loads of the RC columns strengthened with RC jacketing and predicted using the ACI-318 code [43].

Column Shape	Column Specimen	Ultimate Load (kN)	*P*_u.f._ ACI-318 (kN)	Ratio of *P*_u.Ex_/*P*_u.ACI_
Square	C-1	1229	1208	1.02
	C-16	2883	3050	0.95
	C-5	2225	2864	0.78
	C-6	2253	2957	0.76
	C-7	2430	3050	0.80
Rectangular	C-8	1073	1097	0.98
	C-17	2822	3009	0.94
	C-9	2155	2823	0.76
	C-10	2324	2916	0.80
	C-11	2423	3009	0.81
Circular	C-12	635	628	1.01
	C-18	1709	1761	0.97
	C-13	1411	1701	0.83
	C-14	1479	1761	0.84
	C-15	1498	1821	0.82

## Abbreviations

The following abbreviations are used in this manuscript:

*A_c_*	The concrete cross-section area
*A_g_*	The concrete cross-section area inside the stirrups
*A_s_*	The total longitudinal steel reinforcement cross-section
*A_st_*	The steel reinforcement cross-section
*A_sj_*	The cross-section area of steel reinforcement in RC jacketing
*f_c_′*	The concrete compressive strength
*f_y_*	The longitudinal steel yield strength
*P_n.max_*	The design axial load capacity of the RC column
*P_u.Ex_*	The experimental ultimate axial load capacity of the RC column
*P_u.ACI_*	The ultimate axial load capacity of the RC column from the ACI-318 model
*ϕ*	The capacity safety factor
